# Non-uniform self-assembly: On the anisotropic architecture of α-synuclein supra-fibrillar aggregates

**DOI:** 10.1038/s41598-017-06532-1

**Published:** 2017-08-09

**Authors:** Slav A. Semerdzhiev, Volodymyr V. Shvadchak, Vinod Subramaniam, Mireille M. A. E. Claessens

**Affiliations:** 1 Nanobiophysics group, MESA+ Institute for Nanotechnology, University of Twente, P.O. Box 217, 7500 AE Enschede, The Netherlands; 2 Vrije Universiteit Amsterdam, De Boelelaan 1105, 1081 HV Amsterdam, The Netherlands; 3 Institute of Organic Chemistry and Biochemistry AS CR, Prague, 166-10 Czech Republic

## Abstract

Although the function of biopolymer hydrogels in nature depends on structural anisotropy at mesoscopic length scales, the self-assembly of such anisotropic structures *in vitro* is challenging. Here we show that fibrils of the protein α-synuclein spontaneously self-assemble into structurally anisotropic hydrogel particles. While the fibrils in the interior of these supra-fibrillar aggregates (SFAs) are randomly oriented, the fibrils in the periphery prefer to cross neighboring fibrils at high angles. This difference in organization coincides with a significant difference in polarity of the environment in the central and peripheral parts of the SFA. We rationalize the structural anisotropy of SFAs in the light of the observation that αS fibrils bind a substantial amount of counterions. We propose that, with the progress of protein polymerization into fibrils, this binding of counterions changes the ionic environment which triggers a change in fibril organization resulting in anisotropy in the architecture of hydrogel particles.

## Introduction

Under specific physico-chemical conditions, numerous proteins can self-assemble into filamentous aggregates called amyloid fibrils. In the case of the neuronal protein α-synuclein (αS), this phenomenon has been associated with the pathology observed in the brains of patients suffering from Parkinson’s disease^1, 2^. With the onset of this neurodegenerative disorder, αS monomers aggregate into chemically and mechanically stable amyloid fibrils. These fibrils accumulate in the tissue and give rise to morphologically distinct higher order assemblies such as Lewy bodies and Lewy neurites. The formation of amyloids and their accumulation in higher order structures also accompanies the pathology of many other diseases such as Alzheimer’s disease, diabetes type II and Huntington’s disease^3–5^. Besides the importance of amyloids in disease, numerous functional forms of amyloids have been discovered. The existence of functional amyloids, in combination with the chemical and mechanical characteristics of amyloid fibrils, has triggered much interest in using amyloid assemblies as building materials in biomedical and nanotechnological applications^6–12^.

Recent studies showed that αS not only self-assembles into fibrils but depending on the solution conditions it can spontaneously form higher order suprafibrillar aggregates (SFAs). These SFAs are hydrogel particles that, as observed for their *in vivo* counterparts, can adopt different morphologies^13^. Understanding the interactions governing the *in vitro* formation and architecture of these SFAs might thus provide valuable insights into how such structures form *in vivo*. In addition, a better understanding of the organization into suprafibrillar structures may contribute to the knowledge required to manipulate the architecture and properties of new amyloid-based materials.

Here we exploit the observation that αS amyloid fibrils are birefringent to obtain information about the fibril organization in SFAs with a cylinder-like morphology. Birefringence data indicates that the interior and outer parts of the SFA do not share the same fibril organization. Inside the SFAs, fibrils are randomly oriented, rendering the interior of the aggregate optically isotropic. At the periphery, SFAs show clear signs of birefringence indicating a non-random organization of the fibrils in this part of the aggregates. AFM experiments indicate that fibrils at the surface have a preference to cross other fibrils at high angles (close to 90°) probably because this minimizes the repulsive electrostatic interactions between adjacent fibrils. We use a solvatochromic dye with two band emission to sense the polarity of the environment in the different parts of the SFA. This approach confirms the modular design of SFAs and shows different polarity in the interior and the peripheral parts.

We rationalize the difference in fibril organization in the SFA interior and exterior in the context of the observation that amyloid fibrils bind counterions. The aggregation of αS into amyloid fibrils thus results in gradual depletion of counterions. The fibril orientation in the growing SFAs adjusts to these changes, resulting in the formation of cylindrically symmetric anisotropic hydrogel particles.

## Experimental Section

### Materials and methods

#### αS amyloid gels and suprafibrillar aggregates

Expression of the human αS wild-type and the 140 C mutant (αS140C) with a single alanine to cysteine substitution at residue 140, was performed in *E. coli* B121 (DE3) using the pT7–7-based expression system. Details on the purification procedure for αS and αS140C are described elsewhere^14^.

To prepare suprafibrillar αS aggregates 100 µM αS was incubated in 2 mM CaCl_2_ (Sigma), 10 mM Tris (Sigma), pH 7.4, 37 °C for 48 hours. The aggregation was performed in 96 wells plate (Nunc, Thermo Scientific) while shaking at 900 rpm (Heidolph Int., Titramax 100).

#### Polarized light microscopy (PLM)

A glass cell was prepared using a cover slip, glass spacers (1 mm) and a glass slide which were glued together with UV-curable glue. The sample was injected into the glass cell (≈100 µl) with a pipette and was subsequently sealed with vacuum grease. PLM images were acquired with a Leica D/M microscope under crossed polarizers using Leica 20x air objective and recorded with a Leica DFC450C camera.

#### Confocal Laser Scanning Microscopy (CLSM)

The cysteine point mutant αS140C was labelled with AlexaFluor 647 maleimide (Thermofisher scientific, USA) in accordance with the instructions of the manufacturer. SFAs were formed (see material and methods, αS suprafibrillar aggregates) in the presence of 1 mol% of αS140C-Al647. After the SFAs were formed, Thioflavin T (ThT) (Sigma-Aldrich, USA) was added to reach a final concentration of 5 µM. An aliquot was then pipetted into custom made microscopy chambers and imaged on a Nikon Eclipse Ti microscope in confocal laser scanning mode. The ThT and AlexaFluor 647 were excited using 402 nm (CUBE, Coherent Inc., USA). The signal from the ThT dye was collected using a 450/50 nm bandpass emission filter and from the AlexaFluor 647 using a 700/75 nm bandpass filter.

#### Confocal Raman Microscopy (CRM)

For Raman microscopy the SFA containing solution was sandwiched between two coverslips. An airtight seal between these coverslips was created using vacuum grease and an O-ring. Raman images were obtained using a home-built Raman setup at room temperature^15^. A 647.1 nm laser line from a Kr-Ion laser was used to obtain the images. Typically laser powers between 35 mW and 50 mW were used. The images were obtained using integration times of 5 seconds per pixel.

#### Atomic force microscopy

A solution of the SFA was deposited on a mica surface and left at rest for 20 min for the aggregates to sediment and adhere to the substrate. The sample was then gently washed with deionized water (Milli-Q, Milipore Corp., USA) and dried with a nitrogen flow. Height and phase images were obtained using a Multimode 8 AFM (Bruker, USA) in tapping mode and a MSCT Si_3_N_4_ tip (Bruker AFM probes) with expected resonance frequency and spring constant of 85–155 kHz and 0.5 N/m respectively.

#### Fibril organization and cross-angle analysis

The average orientation of the fibrils in the AFM phase images was determined using the ImageJ plugin FibrilTool. Details on how the plugin operates are described elsewhere^16^. Briefly, the program deploys the concept of nematic tensor to identify the average direction of the fibrils and to what extent they are aligned. The tensor is computed on the basis of the pixel intensities in the selected region of interest (ROI). After the intensity gradient is estimated, a unit vector is defined which is a local tangent to the fibrils. The circular average orientation of the unit vector defines the director in the ROI and the circular variance of it determines the degree of ordering (anisotropy).

#### Binding of Co^2+^ by αS fibrils

A 3 mM solution of αS monomers was incubated with fibril seeds at different concentrations of CoCl_2_ (Sigma) for 3 weeks at 37 °C. After that, the aggregated samples were spun down for an hour at 20 000 G. Aliquots of the supernatant were pipetted in a quartz cuvettes and the absorbance at 510 nm (Shimadzu UV-2400PC) was measured in order to spectroscopically determine the concentration of residual (unbound) Co^2+^. The amount of bound Co^2+^ was then calculated and the equilibrium dissociation constant K_d_ was obtained by fitting the found Co^2+^ concentration as a function of the total Co^2+^.

#### Solvatochromic dye imaging

The cysteine point mutant αS140C was first incubated in excess of DTT for an hour. The DTT was removed using a desalting column (Zeba spin, Thermofisher Scientific, USA). Subsequently αS140C was incubated with 4-molar excess of maleimide derivative of 4′-Dimethylamino-3-hydroxyflavone, MFM-maleimide derivative of 1-(2-((2-(4-(dimethylamino)phenyl)-3-hydroxy-4-oxo-4H-chromen-6-yl)amino)ethyl)-1H-pyrrole-2,5-dione) for two hours at room temperature and in the dark^17^. The unreacted dye was removed with a desalting column (Zeba spin, Thermofisher Scientific, USA). SFAs were grown by incubating αS with 5 mol% of αS140C-MFM (100 µM total protein concentration) in identical conditions as described above. The MFM dye was excited using a 402 nm (CUBE, Coherent Inc., USA) laser and the fluorescence from the normal and tautometric forms was collected using 482/35 nm and 595/50 bandpass emission filters respectively.

## Results and Discussion

### Optical properties of αS fibrils

**Figure 1:**
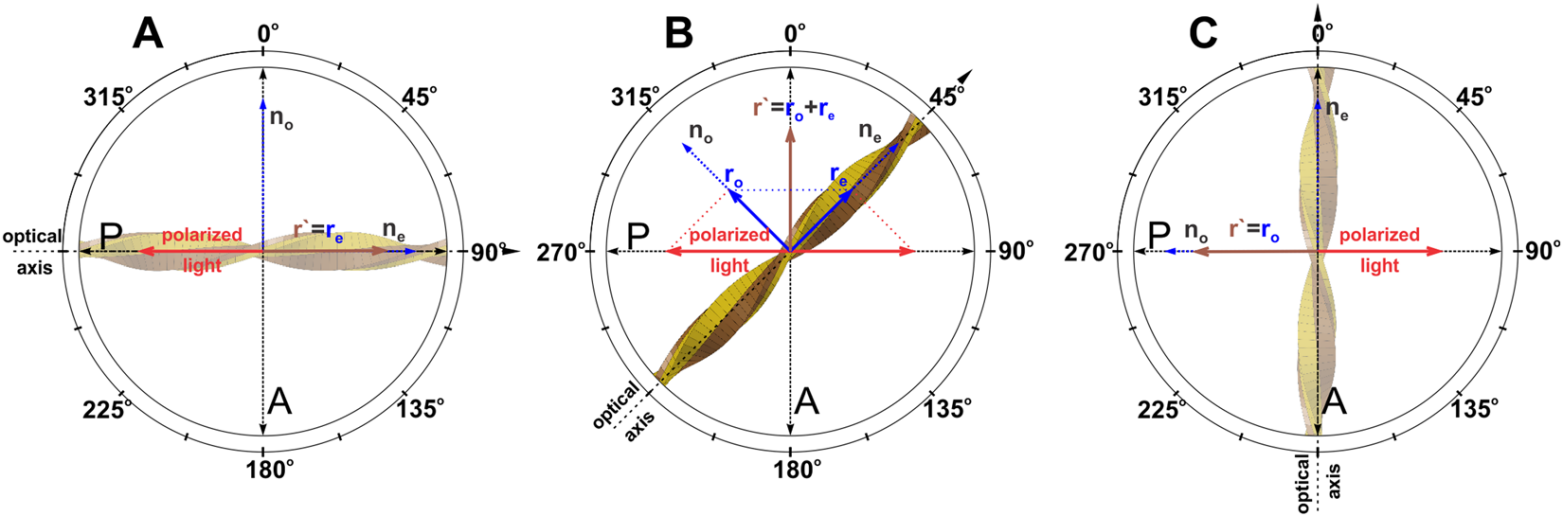
Optical properties of αS fibrils. The blue dotted line vectors represent the ordinary n_o_ (fast) and extra ordinary n_e_ (slow) optical axes of the fibril (yellow). P and A designate the polarizer and analyzer respectively. The blue continuous line vectors and r_e_ show the polarization of the ordinary and extra ordinary rays. r′ is the resultant; the orientation of this vector shows the polarization at which maximum amount of light is transmitted by the fibril, and the length is proportional to the intensity. The red arrows show the polarization of the illumination light. (**A** and **C**) If the optical axis of the fibril stays at an angle that is a multiple of 90° with respect to the polarization axis of A (or of P), the light is completely extinguished r′⊥ A → no transmitted light. (**B**) In contrast, if the angle is a multiple of 45°, maximal amount of light is transmitted r′ = r′_o_ + r_e_.

Proteinaceous materials which exhibit ordering at the molecular level, such as collagen fibers, silk threads and amyloid fibrils, are strongly birefringent upon illumination with polarized light^18, 19^. The polarizability along the backbone of these protein fibers is different from the polarizability at right angles to the backbone which results in different refractive indices for the two directions along the fiber^20^. In amyloid fibrils, proteins (or parts of their sequence) are organized into 1D semi-crystalline arrays of intermolecular cross β-sheets with most of intermolecular hydrogen bonds oriented parallel to the fibril growth axis. Thus, protein monomers stack on top of each other in the longitudinal direction along the fibrils while the backbone of the monomers runs at right angles to it. With this arrangement of protein molecules the optical axis of the fibrils coincides with its longitudinal axis and the refractive index in the lateral and parallel direction should be different (Fig. 1).

### Architecture of the cylindrical SFAs

**Figure 2:**
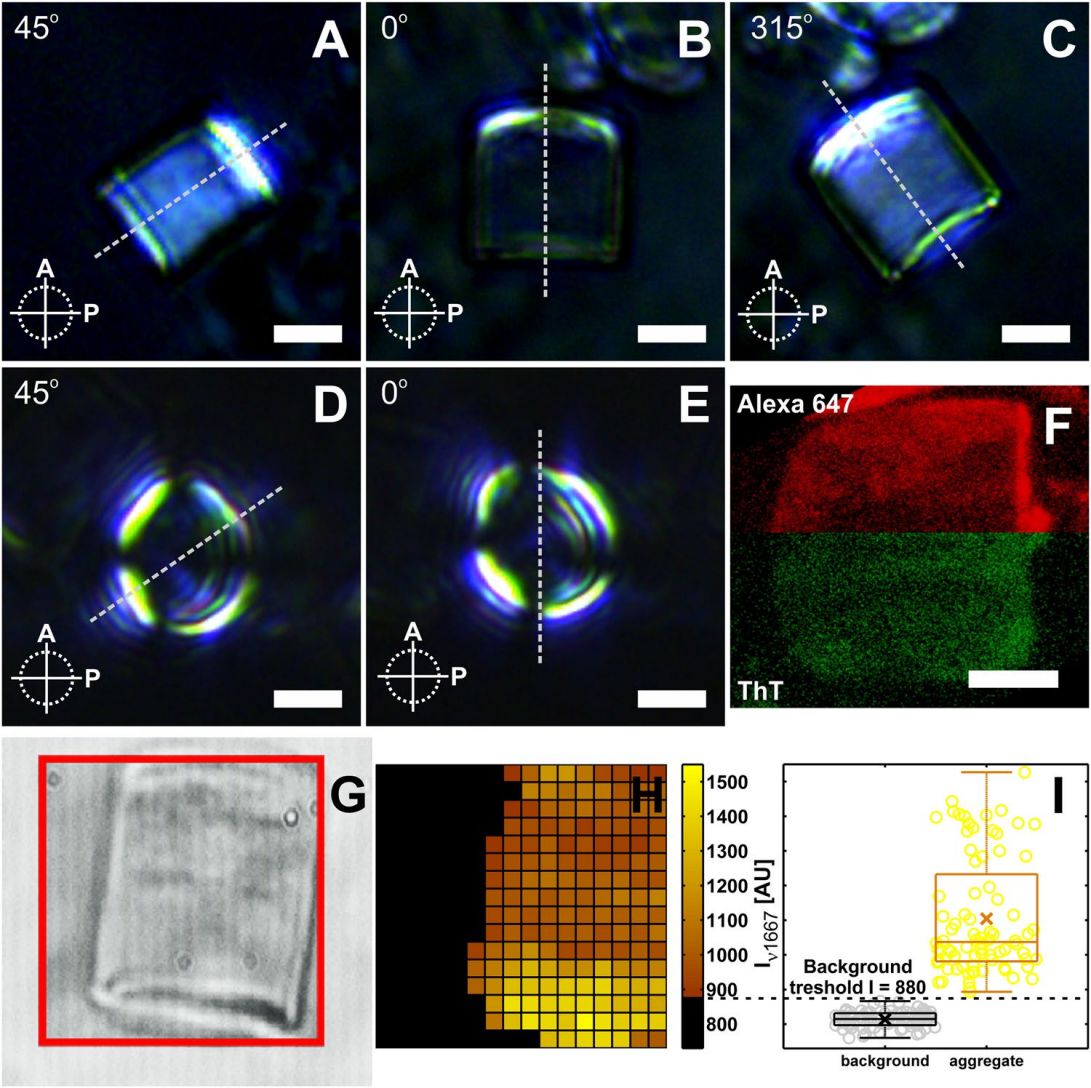
PLM, CLSM and CRM images of SFAs. Side view PLM images of SFA rotated at (**A**) 45°, (**B**) 0° and (**C**) 315° relatively to the polarization axis of the analyzer. Front view PL micrographs of an SFA at (**D**) 45° and (**E**) 0°. (**F**) Confocal z-stack slice, taken along the length of the cylinder, from the middle section of an SFA formed within the presence of αS140C-Al647 (red) and stained with the amyloid specific dye Thioflavin T (green). Scale bars are 10 µm. The dashed line guides the eye for the relative rotation of the aggregate with respect to the crossed polarizers. In the top row (**A**–**C**) the line coincides with the central axis of the aggregates. The red frame in the 40 × 40 μm transmission image (**G**) designates the field of view for the CR mode of the microscope. (**H**) Background pixels which have intensity below the threshold of 880 AU (**I**) are colored in black. All the pixels exceeding the threshold are color coded according to the color scale (above 880 AU) in (**H**).

With the above in mind, it is clear that if the orientation of the fibrils within the aggregates departs from a random distribution the SFA should be birefringent. Upon illuminating the SFAs with plane polarized light and placing them in the right orientation with respect to the crossed polarizers, the birefringent properties of the aggregates become visible (Fig. 2). When viewed from the front the SFAs exhibit a Maltese cross like extinction pattern (Fig. 2D,E). A similar pattern is observed in spherulitic higher order structures of amyloidogenic proteins and other (bio)polymers^21–32^. In spherulites, the Maltese cross pattern is a direct consequence of its molecular architecture. Spherulites consist of fibrils radiating out from a common center. Because of the optical anisotropy of the fibrils and their sphero-symmetric arrangement in the spherulites, the light is completely extinguished at right angles (relative to the polarization axes of the polarizer and analyzer) and attains maximum transmission at 45° (Fig. 1B). However, despite the similarity between the Maltese cross extinction pattern of SFAs and spherulites, there is also a marked difference. While for spherulites the Maltese cross is visible throughout the whole cross-section, in SFAs a large portion of the aggregate remains dark; only the SFA periphery periodically lights up (Fig. 2D,E). The inability of material from the SFA interior to rotate the polarization plane of the light upon transmission can be interpreted in several ways. SFAs with a hollow interior or with an interior composed of amorphously aggregated protein would account for the observed experimental result. Amyloid superstructures with an amorphous core are not unprecedented as it has previously been observed for bovine insulin spherulites^23^. However, the existence of hollow SFAs or SFAs with unstructured cores (no cross-beta sheet content) conflicts with other experimental observations.

**Figure 3:**
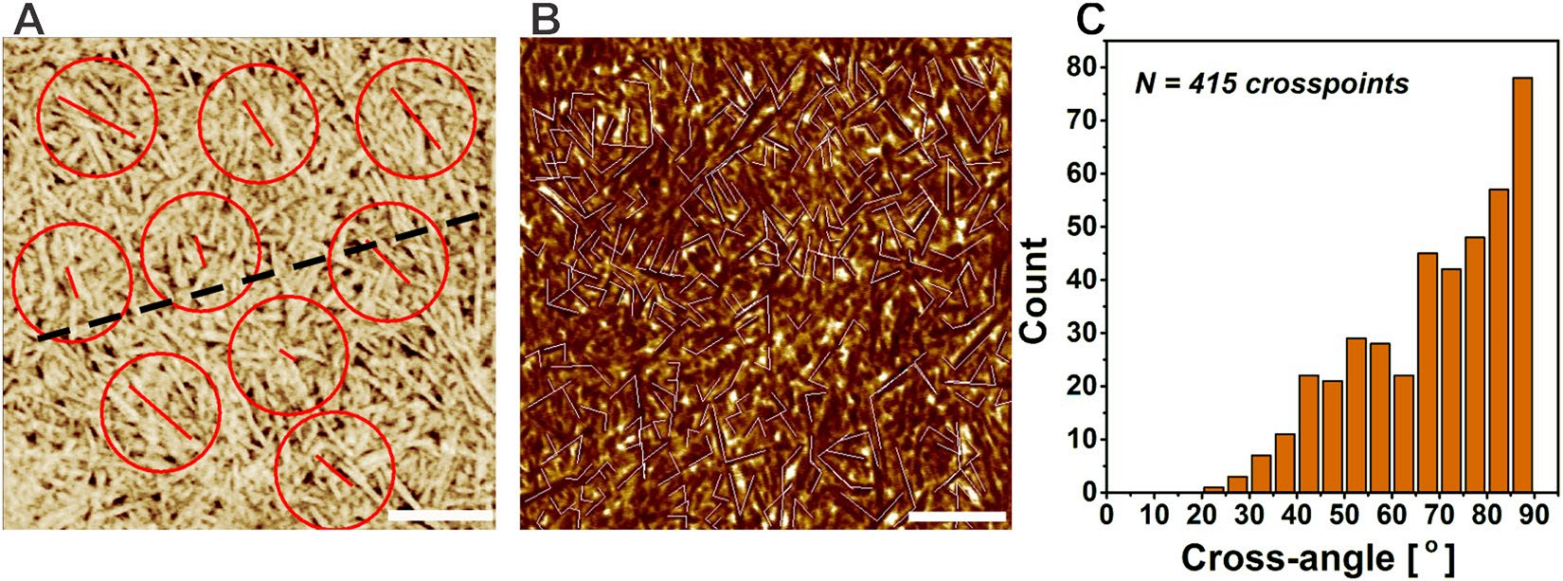
Fibril orientation at the surface of the aggregate. (**A**) AFM phase image from the surface of an SFA. Red circles are the selected areas for which the local director (red lines) was computed. The dashed line represents the central axis of the SFA (a central axis of an SFA can be seen in Fig. 2A–C). (**B**) Representative AFM image with measured fibril cross-angles and (**C**) the corresponding distribution of cross-angles. Scale bars are 500 nm.

Sections of the SFA interior obtained with 3D confocal microscopy clearly show the presence of protein inside the SFAs. The bright Thioflavin T (ThT) fluorescence observed there indicates that these proteins have cross-beta sheet conformation, i.e. they are most probably present in fibrillar form (Fig. 2F). Confocal Raman Microscope (CRM) imaging further supports this finding. The enhanced scattering at the characteristic cross-beta sheet band of ν = 1667 cm^−1^ originating from the interior of the SFAs (Fig. 3G–I) provides additional evidence that the protein is in fibrillar form^33, 34^. Thus, the only explanation that reconciles the data from the confocal and polarized light microscopy is that the core of the aggregates is composed of fibrillar αS and either: (i) all possible orientations of the fibrils have equal weight giving no net director and an optically isotropic material; or (ii) all the fibrils are lying with their optical axis parallel to the long axis of the aggregate. This would cause the light to propagate parallel to the fibrils optical axes (front view Fig. 2D,E) and it therefore experiences no optical anisotropy. If ii) was true then the SFAs should be strongly birefringent when viewed from the side (Fig. 2A–C). Even though the SFAs show birefringence when viewed from the side it is weak compared to the birefringent periphery visible in the front view (Fig. 2). Thus we conclude that the SFAs contain a core of randomly oriented fibrils. Interestingly, the orientation of fibrils changes in the outer layers of the SFAs as is evident from the strongly birefringent periphery of the aggregate when viewed from the front (Fig. 2D,E).

This characteristic extinction pattern does not change upon rotation implying a cylindrical symmetric arrangement of the fibrils in the periphery. To elucidate the source of the strong birefringence from the SFAs periphery, we visualize the fibril arrangement in the outer layers of the aggregates by mapping the surface topology using atomic force microscopy (AFM). It was not possible to visualize individual fibrils on the SFA surface in solution. The SFAs were therefore dried, resulting in a collapse of the hydrogel particles to ~5% of their initial diameter^13^. Subsequently, we analyze the fibril arrangement at the surface in terms of average orientation. This average orientation gives an indication for the source of the observed birefringence. Not surprisingly, the AFM micrographs of the collapsed SFAs do not show large scale order in the fibril arrangement (Fig. 3A). Interestingly, the local directors belonging to the different analyzed surface regions do show a similar orientation. The orientations of the directors often seem close to 45° relative to the longitudinal (central) axis of the SFAs (Fig. 3A). This finding seems to be in conflict with the PLM images. If the fibrils had an average orientation of 45° from the central axis of the aggregates, the maximum transmission of the polarized light would be expected when the central axis of the aggregates approaches 90° (or multiples of it) with respect to the analyzer which is exactly opposite to what is observed (Fig. 2A–C). A possible arrangement of the fibrils that agrees with both the PLM and AFM data is a raft-like structure in which the fibrils cross each other at high angles. If the fibrils have a bimodal preference for a direction such that the optical axis runs either parallel with or perpendicular to the central axis (keeping high angles at the cross-points) of the aggregate, then the SFAs would give maximum transmission of polarized light at positions similar to those observed in Fig. 2A and C. Collecting the distribution of angles at which fibrils cross each other on the surface of the SFAs results in a histogram that peaks at high angles which is in line with the proposed preferential arrangement of the fibrils. It should be noted that this arrangement is also in line with the extinction patterns that are observed when the SFAs are imaged from the front (Fig. 2D,E). If one assumes that the outer layers of the aggregates are composed of stacks of 2D fibrils rafts or meshes, then it is easy to explain the extinction pattern observed in front view PLM images of SFAs. There are two points that need to be considered to explain the birefringence observed in front views of αS SFAs. One is that to get birefringence the fibril’s optical axis should lie in an orientation that is not parallel to the direction in which the polarized light propagates and the second is that for birefringence to occur the slow and fast axes of the fibril should be at an angle different from 0°, 90°, 180° or 270° with respect to the analyzer (Fig. 1). Since, there will always be a large fraction of fibrils in the rafts that do not align in parallel with the central axis of the SFA, the first condition is essentially fulfilled for the whole periphery of the aggregate (2π). That does not hold true for the second condition. The position of the fibrils confined in the 2D mesh fails to comply with the second condition at the top, bottom and the sides of the SFA periphery (when looking from the front) leaving those areas dark (Fig. 2D,E) under the cross polarizers resulting in the Maltese cross like extinction pattern.

**Figure 4:**
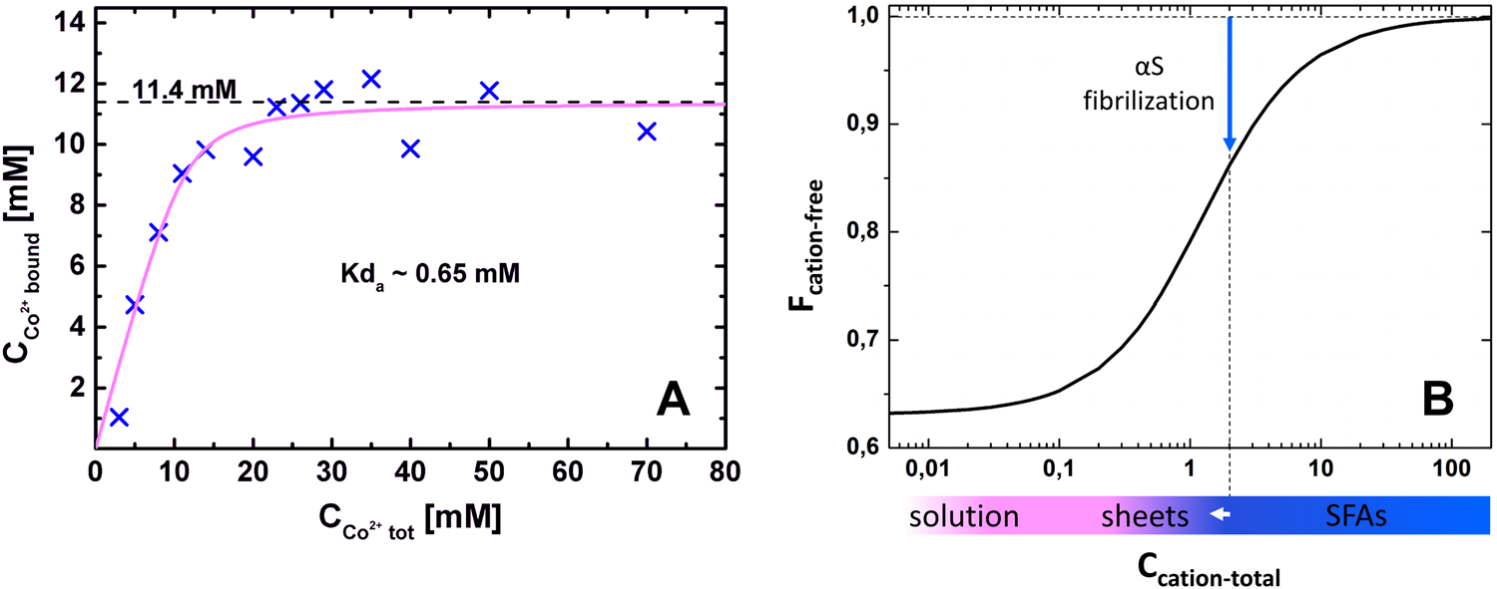
Co^2+^ binding by αS. (**A**) Binding of Co^2+^ ions by αS. (**B**) Calculated fraction of divalent cations that remain in solution as a function of the total divalent cation concentration at 100 µM αS assuming K_d_ = 0.65 mM (**A**). At the experimental conditions used here to produce SFAs (2mM CaCl_2_) a significant fraction of the cations is expected to become associated with fibrils (vertical blue arrow). The higher order organization of fibrils is very sensitive to ionic strength of the solution. The horizontal bar indicates the transition from a solution of fibrils (white) to fibrillar sheets (pink) and SFAs (blue). Since at 2 mM a significant fraction of the cations is depleted from solution during polymerization (and SFA formation) the solution conditions move closer to the sheet-SFA transition point and possibly cross it (horizontal white arrow). In response to that the newly formed outer layers of the SFA adopt a different architecture where the fibril mutual orientation converges towards higher crossing angles.

**Figure 5:**
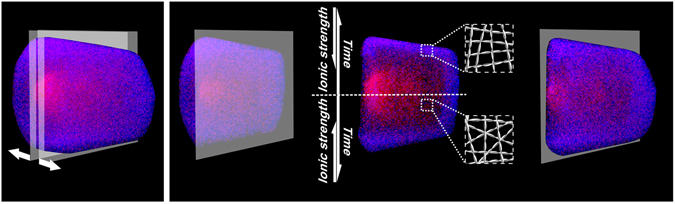
Artistic impression on the internal architecture of SFAs formed at initial ionic strength of 2mM CaCl_2_. (**A**) A 3D CLSM image of a SFA. (**B**) Proposed fibril organization mapped on a lateral slice of the SFA. In time, new sections of the SFA are formed while the “effective” ionic strength is reduced due to the recruitment of counterions by the fibrils incorporated in the SFAs.

A non-random organization of fibrils confined at the surface of a cylinder is not unusual for semi-flexible (bio)polymers. Cellulose fibrils wrap the plant cell walls in a helical manner^35, 36^. Similarly, collagen fibers twist around blood vessels following a helical trajectory and interestingly in some cases - including the thoracic aorta and annulus fibrosus - a cross-hatched order of the filaments is observed^37–40^. It has been previously reported that strain may induce the alignment of semiflexible polymers^41^. A helical organization of semiflexible polymers confined at the surface of carbon nanotubes has been also observed in simulations^42, 43^. Recent simulations with 2D confined Mikado networks on cylindrical substrates showed a spontaneous bimodal orientation of the cross-linked filaments imposed by the interplay between stretching and bending energies of the individual polymers^44^. This bimodal orientation of fibrils confined on cylindrical surfaces is reminiscent of the arrangement of αS fibrils in the periphery of the SFAs. The mechanisms that are responsible for the orientation of fibrils confined on cylinders may therefore possibly also be used to rationalize fibril orientation in SFAs. This cross-hatch model however, predicts a bimodal distribution of orientations centered around ±π/4 with respect to the central axis of the cylinder, which disagrees with the PLM images. Moreover, the radius of curvature of the SFA is rather large with respect to the fibril persistence length, which makes the energy associated with fibril bending relatively low. In addition, the inter-fibril cross-links are transient which allows the fibrils to readjust and control the build-up of stresses and minimize fibril stretching. Thus, the role of stretching and bending fibrils in imposing order in the periphery of SFAs is probably small. Instead inter-fibril electrostatic interactions may be the force driving the high angle fibril arrangement. αS fibrils are net highly negatively charged, high cross-angles are therefore favored over parallel fibril orientation since they minimize the electrostatic repulsion. Electrostatic interactions may thus be responsible for the observed distribution of the fibril cross-angles at the surface of the SFAs (Fig. 3C). A similar arrangement of filaments has been observed in supramolecular raft assemblies formed by the negatively charged biopolymer f-actin^45^. In the presence of divalent counterions, including Ca^2+^, f-actin assembles into ion cross-linked lamellar rafts. In this system the actin filaments are arranged at high angles to diminish the electrostatic repulsion. Interestingly, for f-actin rafts the Ca^2+^ concentration at which the transition from an isotropic to a ‘raft-like’ phase takes place is very close to the Ca^2+^ concentration used to form the αS SFAs. At the relatively low Ca^2+^ concentrations used to induce the formation of SFAs, the number of condensed Ca^2+^ might not be sufficient to neutralize the charge of the αS fibrils. As a result of the relatively high fibril charge density fibrils prefer high angle crossings over parallel arrangements. However, although Ca^2+^ does not neutralize the fibril charge, its concentration is high enough to establish cross-links between αS fibrils at the intersection points. This explains the orientation of fibrils at the surface of the SFAs but it still remains unclear what triggers the change in the organization of the fibrils at the peripheral layers of the SFA compared to the interior. To obtain an anisotropic fibril organization some dynamic parameter should feed back into the self-assembly process. It has been proposed previously that the strength of the electrostatic interactions can determine the morphology of protein aggregates^46^. This offered a plausible explanation for the polymorphism that is experimentally observed among SFAs of different proteins. In particular it could account for the structural anisotropy of the insulin spherulite core and periphery. There it was argued that the accumulation of repulsive electrostatic interaction forces causes the developing aggregates to change their fractal dimension^46^. The internal architecture of the spherulite changes in order to compensate for energy build-up. Perhaps a similar reasoning can be applied for the αS SFAs. The proposed mechanism would agree with previous findings showing that the growth of SFAs can be reinitiated or stopped when the ionic strength of the solution is increased or decreased respectively^13^. An alternative or complementary mechanism that could be considered is based on the change in availability of counterions. Experiments show that due to their surface charge αS (and other proteins) amyloid fibrils bind a significant amount of multivalent counterions (Fig. 4)^9^. Thus, in the course of their development, SFAs deplete the solution of a significant amount of counterions which decreases the bulk concentration of ‘free’ ions (Fig. 4). This has two consequences: (i) in time the charge screening deteriorates and the new layers of the SFA form in this changed environment; (ii) the amount of condensed ions on the new fibril layers is lower which reduces the charge neutralization and the ‘cross-linking’ that the Ca^2+^ provides at the fibril intersection points. The decrease in counterion concentration in solution may thus result in a change in the structure of the SFA. This interpretation agrees with the theoretical predictions for the phase behavior of semi-flexible polymers in the presence of cross-linkers. At high cross-linker concentration, a gel is expected to be the equilibrium phase while at sufficiently low concentrations, raft-like structures are predicted to form ref. 47. Thus the conditions at the first stages of SFAs formation might favor a gel-like interior while at a later stage, at which the cross-linkers are more scarce, a raft-like architecture may be preferred (Fig. 5). Changes in ionic environment might thus give rise to the modular architecture observed for fibrils in the SFAs. The sensitivity of the αS hierarchical self-assembly to the ionic strength of the solution is very well demonstrated by the changing morphology of the SFAs at different CaCl_2_ concentrations^13^. In the experiments presented here, the SFAs were formed at 2 mM CaCl_2_, just above the reported transition from fibrillar sheets to cylindrical SFAs. The proximity of the experimental conditions to this transition point indicates that the fibril organization is very sensitive to changes in the ionic environment that may occur during aggregation of αS into fibrils. The observed significant depletion of counterions thus provides supporting evidence for the proposed explanation regarding the changing architecture of the SFAs formed at 2 mM CaCl_2_ (Fig. 4).

### Polarity map of cylindrical SFAs

**Figure 6:**
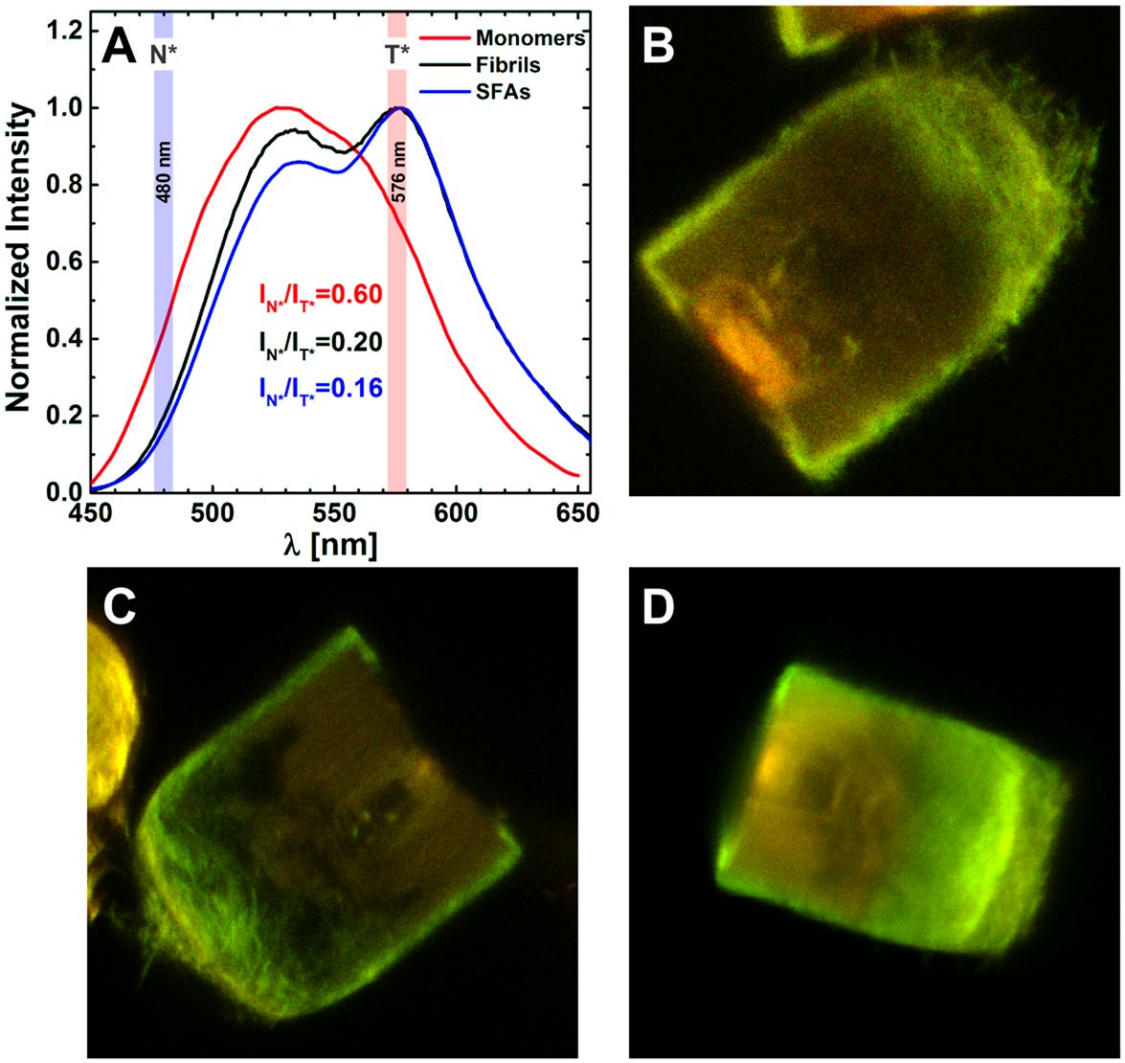
(**A**) Normalized fluorescence emission spectra for aS140C-MFM in monomeric form and in different aggregation states. The environment becomes more polar in the order monomer < fibril < SFAs. Intensities for the N* and T* bands were measured at 480 nm and 576 nm respectively. (**B**–**D**) CLSM images (red and green overlay) of suprafibrillar aggregates containing 5% αS140C-MFM. The green and red color correspond to emission of the N* and T* forms of the dye recorded at 482 nm (bw 35 nm) and 595 nm (bw 50 nm) respectively. More green color in the image corresponds to regions of more polar environment in the SFAs. The intensity is proportional to the fibril density.

The modular architecture of the SFAs is very interesting but at the same time very surprising. To further investigate the structure of the SFAs we apply a novel approach and use the properties of a solvatochromic dye to sense the environment polarity within the different regions of the SFAs. 3-hydroxyflavone fluorophores can undergo an excited state intramolecular proton transfer (ESIPT) which generates a second tautomeric (T*) band in the emission spectrum (Fig. 6A)^48^. The intensity ratio of the normal (N*) and tautomeric band is highly sensitive to the local environment and increases in more protic and polar environments^49, 50^. Because of these features this class of dyes permits the spectroscopic discrimination between different membrane types^50–52^. Also, 4′-diethylamino-3-hydroxyflavone has been successfully used to probe differences in supramolecular organization of amyloid fibrils^53^. Another ESIPT probe (MFC) has been used to monitor αS aggregation^54^. Here we use the MFM label, a maleimide derivative of the 4′-dimethylamino-3-hydroxyflavone fluorophore, to determine if the difference in fibril organization in SFAs also results in differences in physico-chemical properties of the fibril environment. First, we investigate if MFM is able to differentiate the aggregation states of αS. To that end we prepared αS fibrils and SFAs containing 5% of αS140C mutant labeled with MFM (αS 140C-MFM) and compared the emission of the dye in such aggregates with the emission in fibrillar and monomeric αS. A marked difference in the emission spectra can be observed for the different states of the protein (Fig. 6A). As anticipated, the fluorophore detects a decrease in polarity of its environment in the order monomers > fibrils > SFAs (Fig. 6A). Attached to monomeric αS, MFM exhibits a single N* band corresponding to the dye emission in highly polar (hydrated) environment. In fibrils the environment is more hydrophobic and the band of the T* form appears in the emission spectrum of MFM label (Fig. 6A). The relative intensity of the T* band increases even more once SFAs are formed.

**Figure 7:**
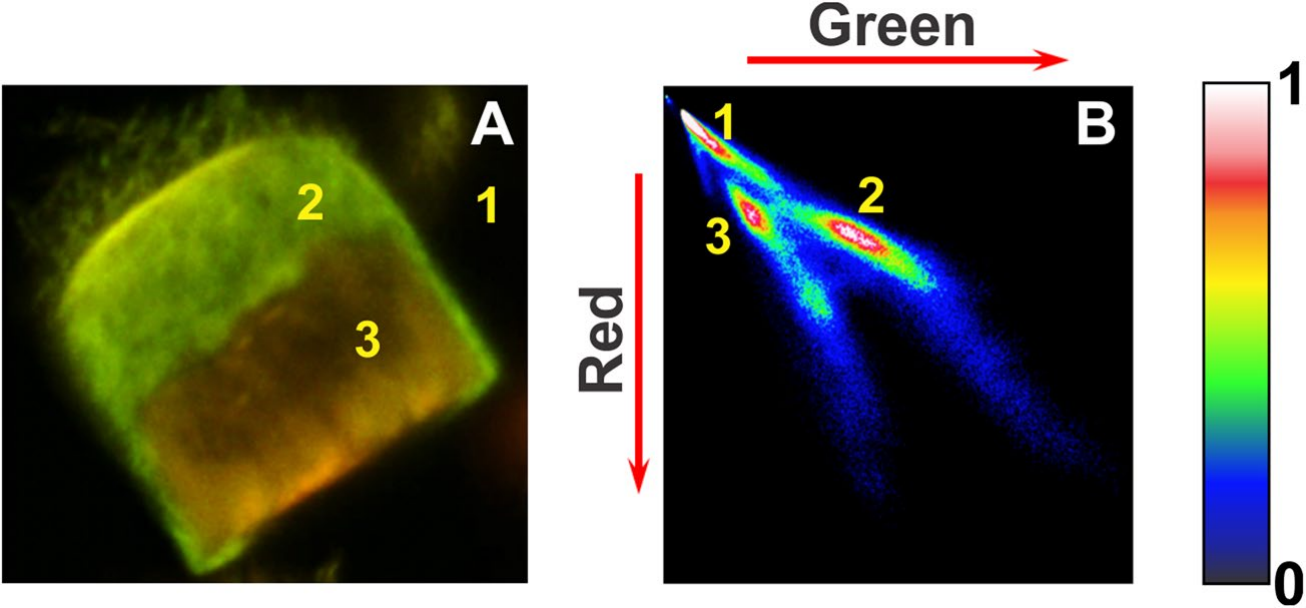
Distribution analysis for aS140C-MFM in SFAs. (**A**) CLSM image of an SFA that is analyzed. (**B**) Red intensity vs Green intensity contour map. Populations 1, 2, 3 correspond to the background, more polar external part of SFA, and more apolar internal part of SFA respectively.

That points to a less polar environment and therefore more dense protein packing in the SFAs compared to that in individual fibrils suspended in the solution. Since the emission intensity ratio of the short and long wavelength bands of MFM can report differences in the average environment polarity in fibrillar and SFA form of αS, it is possible that the dye is sensitive enough to detect differences in the physico-chemical environment inside the SFAs. To put that conjecture to the test, we have acquired CLSM images of SFAs that were formed in the presence of 5% of αS140C-MFM (Fig. 6). Difference in the ratio of the intensity of the N* and T* forms of the MFM on these images shows that the outer layers of the SFAs are more polar than the inner parts. Interestingly, the polarity pattern outlines a modular design that is strongly reminiscent of the architecture deduced from the PLM images. Since, the interior and the periphery of the SFAs are comprised of fibrils, most likely their arrangement and/or packing (i.e. density) causes the different behavior of the dye localized in the different parts of the aggregate. However, the ratio between the T* and N* signal remains constant despite the increase in the intensity of both (Fig. 7). That holds for the interior and the peripheral part of the SFAs as can be seen by the two populations in the contour plots (Fig. 7). This observation excludes that variations in fibril density are responsible for the changes in polarity. Differences in fibril arrangement remain a potential explanation for the observed spectroscopic behavior of the solvatochromic dye incorporated in different regions of the SFAs.

**Figure 8:**
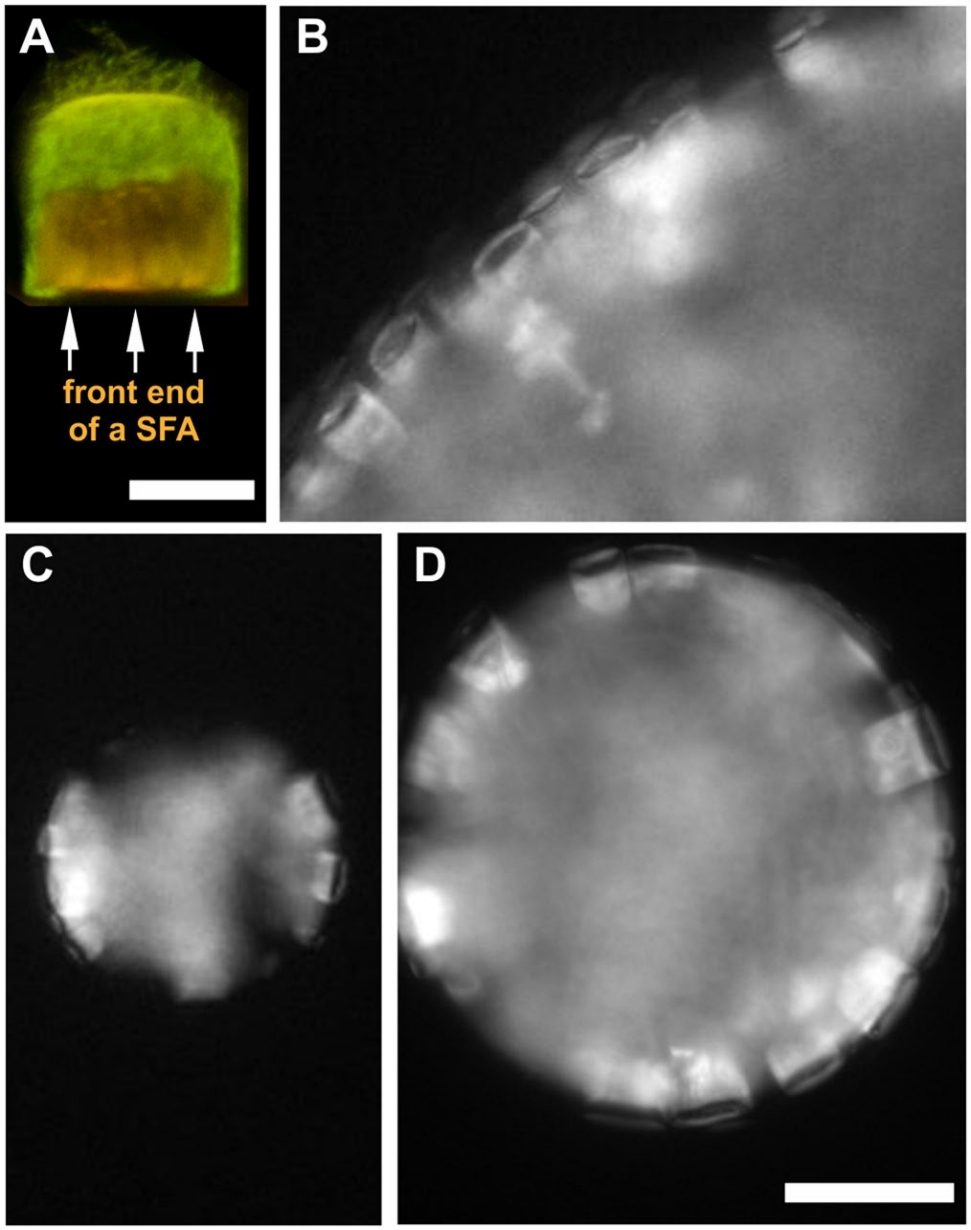
Alignment of αS SFAs at oil/water interface. (**A**) Ratiometric confocal fluorescence image of an αS SFA containing aS140C-MFM. The ratiometric image indicates that the outer layers of the SFAs are more polar than the inner parts. (**B**–**D**) Epi-fluorescence images of SFAs prepared in 10 mM Tris, 2 mM CaCl_2_ and 100 μM αS stained with ThT and re-suspended in 2 vol% water/n-dodecane emulsion. Under these conditions the SFAs localize in the water phase but remain in contact with the oil phase by exposing their front parts to it. This indicates a more apolar interior of the aggregate. At the front of the SFAs a large part of the interior is exposed to the solvent (shown in **A**). Scale bars are 25 μm (**A**) and 100 μm (**D**).

Thus, we rationalize these observations in the context of the proposed difference in organization of the fibrils in the periphery and interior of the hydrogel aggregates. The size of contact area between fibrils is strongly dependent on their mutual orientation. Thus, if the angles are higher – as is expected for the interior of the SFAs - the interfibril contact area inside the aggregates will be large compared to the contact area of fibrils in the SFA periphery. We hypothesize that at higher interfibril angles, more of the unstructured C-terminal regions on the fibril surface (and therefore dye molecules) become ‘trapped’ between the two interfacing hydrophobic surfaces of the fibril cores. As a result, the dyes coupled to the C-termini experience a more apolar environment. Since it is expected that number of trapped C-termini are higher inside the SFAs than at their periphery, we postulate that this leads to a lower *I*_N*_ */I*_T*_ for the signal originating from the interior of the SFAs. Additionally, the degree of collapse of the C-terminal region may differ in the different parts of the SFAs. As mentioned earlier, because of the recruitment of counterions by the fibrils, the outer parts of the aggregates effectively form at different ionic strengths. Thus, C-terminal regions with higher degree of collapse (interior of SFAs) effectively shorten the average distance between the hydrophobic core of the fibril and the last residue of the protruding C-termini of the monomers. The collapse of the C-terminus may also increase the exposure of the hydrophobic core of the fibrils rendering the overall environment in the interior of the SFAs more hydrophobic (Fig. 8). Thus, the aforementioned two effects probably both contribute to a lower *I*_N*_ */I*_T*_ for the signal emanating from the interior of the aggregates.

## Conclusion

αS amyloid fibrils are, similar to fibrils of other amyloidogenic proteins, birefringent. We have exploited this optical property to probe the architecture of SFAs and have visualized the modular build-up of these structures, emanating from the different organization of the fibrils in the interior and periphery of the SFAs. The fibrils located in the internal parts of the aggregates have a random distribution of orientations while the fibrils at the periphery preferably cross at high angles. We rationalize these findings in the light of the observation that αS fibrils bind divalent ions. While at the early stages of the self-assembly phenomenon SFAs form in a counterion “rich” environment, with the progression of the aggregation process increasing amount of counterions become trapped by the newly formed fibrils. Thus, at later stages, SFA growth continues in an environment with a lower ion concentration. This change in the ionic environment feeds back into the fibril organization of the growing suprafibrillar aggregates. As a result of the decrease in the free counterion concentration higher crossing angles between fibrils are established to minimize the electrostatic repulsion and allow the aggregates to grow further. A novel approach based on the conjugation of a solvatochromic dye with αS monomer reveals that the change in fibril organization results in the formation of regions with different polarity. The outline of the regions visualized using the solvatochromic dye coincides remarkably well with SFA structural design derived from PLM images. This allow us to hypothesize that the difference in polarity sensed by the MFM dye is imposed by the different organization of the fibrils in the interior and peripheral parts of the hydrogel aggregates. In the interior, where the orientation of fibrils is isotropic and low interfibril cross-angles are more probable, a larger fraction of C-terminal regions may find themselves trapped between the cores of adjacent fibrils at the crossing points. This renders the environment that the MFM dye – which is coupled to the C-terminus of the protein - experiences inside the aggregate more apolar then the environment in the outer layers of the SFA where higher inter-fibril angles are preferred.

The architectural switch within the SFAs gives rise to particles with unique structural anisotropy, a feature that is in general difficult to achieve for finite sized colloidal objects without a complicated synthesis strategy. The findings in this work could act as a source of inspiration for a novel and relatively simple strategy to synthesize structurally inhomogeneous proteinaceous particles which could be potentially interesting for numerous fields of application. For example, such particles could be easily deployed as carriers for apolar compounds which need to be suspended in aqueous solution.

The observed structural anisotropy within the SFAs is also somewhat reminiscent of the characteristics of pathological fibrillar aggregates such as Lewy bodies. The core of such *in vivo* aggregates is more densely packed while the periphery appears as a loose halo. Considering that at physiological αS concentrations (20–40 µM), both the Mg^2+^ and K^+^ concentrations are in the regime where we observe a transition from sheets to SFAs, the sensitivity of αS self-assembly to the physico-chemical conditions that is responsible for the anisotropy of *in vitro* formed SFAs could also be the cause of the anisotropy observed in their *in vivo* counterparts. The onset of disease may trigger changes in the physico-chemical conditions within the cell. These changes may in turn trigger an adaptation of the architecture of the parts of the aggregates that were formed at different time points/intervals.
